# Lactate is a potent inhibitor of the capsaicin receptor TRPV1

**DOI:** 10.1038/srep36740

**Published:** 2016-11-09

**Authors:** Jeanne de la Roche, Isabella Walther, Waleria Leonow, Axel Hage, Mirjam Eberhardt, Martin Fischer, Peter W. Reeh, Susanne Sauer, Andreas Leffler

**Affiliations:** 1 Department for Anaesthesiology and Critical Care Medicine, Hannover Medical School, Carl-Neuberg-Str.1, 30625 Hannover, Germany; 2 Institute for Neurophysiology, Hannover Medical School, Carl-Neuberg-Str.1, 30625 Hannover, Germany; 3 Institute of Physiology and Pathophysiology, Friedrich-Alexander-University Erlangen-Nuremberg, 91054 Erlangen, Germany

## Abstract

Tissue ischemia results in an accumulation of lactate and local or systemic lactic acidosis. In nociceptive sensory neurons, lactate was reported to sensitize or activate the transient receptor potential ion channel TRPA1 and acid-sensing ion channels (ASICs). However, it is unclear how lactate modulates the TRPV1 regarded as the main sensor for acidosis in sensory neurons. In this study we investigated the effects of lactate (LA) on recombinant and native TRPV1 channels and on TRPV1-mediated release of neuropeptides from mouse nerves. TRPV1-mediated membrane currents evoked by protons, capsaicin or heat are inhibited by LA at concentrations ranging from 3 μM to 100 mM. LA inhibits TRPV1-mediated proton-induced Ca^2+^-influx in dorsal root ganglion neurons as well as proton-evoked neuropeptide release from mouse nerves. Inhibition of TRPV1 by LA is significantly stronger on inward currents as compared to outward currents since LA affects channel gating, shifting the activation curve towards more positive potentials. The mutation I680A in the pore lower gate displays no LA inhibition. Cell-attached as well as excised inside- and outside-out patches suggest an interaction through an extracellular binding site. In conclusion, our data demonstrate that lactate at physiologically relevant concentrations is a potent endogenous inhibitor of TRPV1.

Sensory neurons are equipped with numerous specialized membrane proteins which mediate the sensation of pain when challenged by tissue injury, inflammation and metabolic disturbances^1^. Protons gate or modulate several membrane receptors in nociceptive sensory neurons, and studies on transgenic mice lacking acid sensing ion channels (ASICs) or the transient receptor potential vanilloid 1 (TRPV1) cation channel have shown the significance of these transduction molecules for acid-sensing of sensory neurons^2^,^3^,^4^,^5^. While proton sensitivity of unmyelinated C-fibers in mice seems to depend on TRPV1^2^,^6^, pharmacological experiments found that ASICs rather than TRPV1 mediate proton-induced pain in human skin^7^,^8^. In addition, we recently reported that the human isoform of the transient receptor potential ankyrin 1 (hTRPA1) cation channel is activated by extracellular protons^9^.

Tissue acidosis associated with ischemia is caused by an excessive accumulation of lactate (LA) with local concentrations up to 50 mM^10^,^11^,^12^. Furthermore, acidosis due to tissue inflammation involves the release of lactate from bacteria and immune cells^13^. While most *in vitro* studies exploring acid sensitivity of sensory neurons were performed with buffered acidic solutions lacking lactate, a limited number of studies suggest that both ASICs and TRPA1 distinguish between acidosis and lactic acidosis. LA sensitizes ASICs by acting as a chelator for divalent cations^14^. TRPA1 was recently reported to be the principle molecule for activation of sensory neurons by weak acids^15^. While both rodent and human TRPA1 are activated by intracellular acidosis, only human TRPA1 is activated by externally applied protons^9^.

Previous publications only indirectly indicate LA effects on TRPV1. LA was reported to potentiate proton-evoked release of calcitonin gene-related peptide (CGRP) from rat spinal cord slices^16^. As later studies demonstrated that proton-evoked release of CGRP from sensory neurons crucially depends on TRPV1^17^, this result indicates that LA might also potentiate TRPV1. On the other hand, weak acids were shown to block TRPV1^15^. Furthermore, protons inhibit TRPV1 by interfering with permeation of cations^18^,^19^,^20^. As LA induces intracellular acidosis, the existing literature indicates that LA should inhibit TRPV1.

In this study we demonstrate that LA inhibits TRPV1 channels from the extracellular side and independently of intracellular acidosis. Patch clamp and calcium imaging were employed to investigate the effects of LA on gating of recombinant wild type and mutant TRPV1 constructs, and also on TRPV1 in mouse dorsal root ganglion neurons. Furthermore, we performed enzyme linked immunosorbent assay to explore the effects of LA on TRPV1-mediated CGRP-release from isolated mouse sciatic nerves.

## Results

### Lactate inhibits proton-evoked activation of TRPV1

**Figure 1:**
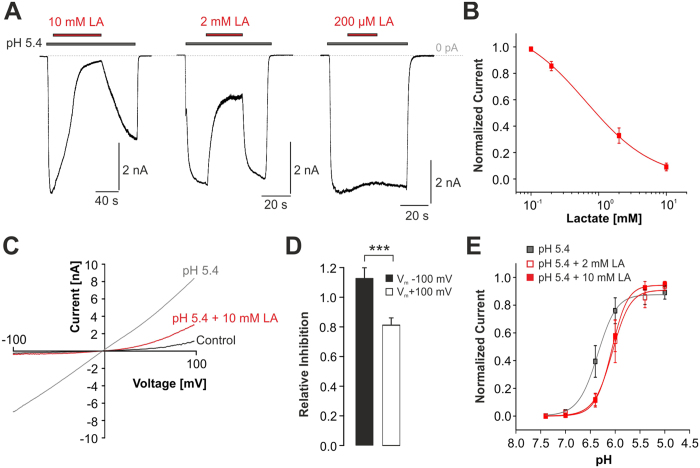
Inhibition of proton-evoked TRPV1 currents by LA. (**A**) Representative recordings demonstrating inhibition of a pH 5.4-induced inward current by LA at different concentrations. Cells were held at −60 mV and the co-application of LA and pH 5.4 was started once the pH 5.4-induced inward current had reached a steady-state. (**B**) Concentration-dependency of LA-induced inhibition of proton-evoked inward currents. Current amplitudes were normalized to those before LA application. The solid line represents a fit with the Hill equation. (**C**) Membrane currents of TRPV1 monitored during 500-ms long voltage ramps from −100 mV to +100 mV in presence of control solution, pH 5.4 or pH 5.4 + 10 mM LA. (**D**) Mean relative inhibition of pH 5.4-induced inward currents at −100 mV and outward currents at +100 mV by 10 mM LA. The average relative inhibition = 1 − I/I_0_ for each concentration was determined by normalization; where I = current in presence of LA, I_0_ = current in absence of LA. Inward currents were significantly stronger inhibited as compared to outward currents. (**E**) Concentration-dependent activation of TRPV1 by protons applied alone or in combination with 2 or 10 mM LA. Peak current amplitudes were normalized to the largest current amplitude in the respective cells and plotted against the corresponding pH-values. The solid lines represent fits with the Hill equation. Data are presented as mean ± S.E.M. Statistical differences are indicated by ***p < 0.001.

We first examined the effects of different concentrations of LA on proton-evoked activation of human TRPV1 expressed in human embryonic kidney cells 293T (HEK293T). When TRPV1 was activated by extracellular pH 5.4 at a holding potential of −60 mV, co-application of 10 mM LA buffered to pH 5.4 resulted in a prominent and partly reversible inhibition of the inward current (91 ± 3% inhibition, n = 7; Fig. 1A). While such high concentrations of LA accumulate in tissue under ischemic conditions^10^,^11^,^12^, we next explored the effects of lower and thus physiologically more relevant concentrations of LA on proton-evoked inward currents (Fig. 1A). As is demonstrated in Fig. 1B, the IC_50_-value for LA-induced inhibition of pH5.4-evoked inward currents was calculated to 0.7 ± 0.1 mM (n = 5–7 for each concentration, Hill coefficient 0.9 ± 0.07). Thus at 2 mM, closely matching the physiological plasma level of LA in healthy subjects, LA seems to induce a substantial inhibition of TRPV1. We next explored if LA inhibits TRPV1 in a voltage-dependent manner. Membrane currents were induced by pH 5.4 and monitored during a 500 ms long voltage ramp with a linear increase from −100 to +100 mV (Fig. 1C). The inhibitory effect of 10 mM on inward currents at −100 mV reached 112 ± 7% (n = 6), i.e. the remaining current with pH 5.4 and 10 mM LA was smaller than the leak current determined prior to the experiment. However, inhibition of outward currents at +100 mV was significantly smaller (80 ± 5%, n = 6; p < 0.001, paired t-test; Fig. 1D). As TRPV1 probably acts as an important proton sensor in sensory neurons, we also asked if LA impairs the proton-sensitivity of TRPV1 and modulates the IC_50_-value for proton-evoked activation. In absence of LA, protons activated TRPV1 with an EC_50_-value of pH 6.4 ± 0.02 (n = 7). In presence of 2 mM or 10 mM LA, we observed a non-significant shift of the dose-response curve (Fig. 1E; 2 mM LA: EC_50_ pH 6.1 ± 0.02, n = 7; 10 mM LA: EC_50_ pH 6.1 ± 0.01, n = 7).

### Lactate inhibits TRPV1-mediated membrane currents evoked by different agonists at pH 7.4

**Figure 2:**
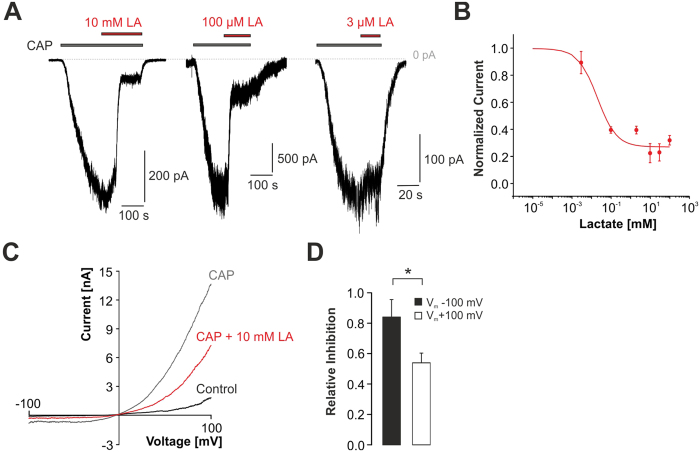
Inhibition of capsaicin-evoked TRPV1 currents by LA. (**A**) Representative recordings demonstrating inhibition of a capsaicin-induced inward current by different concentrations of LA. Cells were held at −60 mV and the co-application of LA and capsaicin (CAP, 50 nM) was started once the capsaicin-induced inward current had reached a steady-state. (**B**) Concentration-dependency of LA-induced inhibition of capsaicin-evoked inward currents. Current amplitudes were normalized to those before LA application. The solid line represents a fit with the Hill equation. (**C**) Membrane currents of TRPV1 monitored during 500-ms long voltage ramps from −100 mV to +100 mV in presence of control solution, capsaicin or capsaicin +10 mM LA. (**D**) Mean relative inhibition of capsaicin-induced inward currents at −100 mV and outward currents at +100 mV by 10 mM LA. Inward currents were significantly stronger inhibited as compared to outward currents. Data are presented as mean ± S.E.M. Statistical differences are indicated by *p < 0.05.

We next explored if LA inhibits activation of TRPV1 by capsaicin (CAP) or heat at neutral pH-values (pH 7.4). Considering that LA is a weak acid causing substantial acidosis upon accumulation, high concentrations (>2 mM) of LA at neutral pH-values do not occur *in vivo*. Taking into regard that a substantial amount of LA only permeates the membrane at acidic solutions, we assume that effects induced by extracellularly applied LA at pH 7.4 are not due to induction of intracellular acidosis. As is demonstrated in Fig. 2A,B, capsaicin-induced inward currents were indeed inhibited by LA in a concentration-dependent manner. The IC_50_-value for this LA-induced inhibition was 20 ± 12 μM (n = 4–6 for each concentration), thus considerably lower than the value obtained for proton-evoked currents. Similar to proton-evoked membrane currents, capsaicin-induced inward currents at −100 mV (85 ± 12%) were significantly stronger inhibited by 10 mM LA as compared to outward currents at +100 mV (54 ± 6%, n = 6; p < 0.05, paired t-test; Fig. 2D). In contrast to proton-evoked currents which were almost completely inhibited by 10 mM LA, capsaicin-induced currents were less affected by concentrations up to 100 mM (100 mM LA: 63 ± 8% inhibition, n = 7).

**Figure 3:**
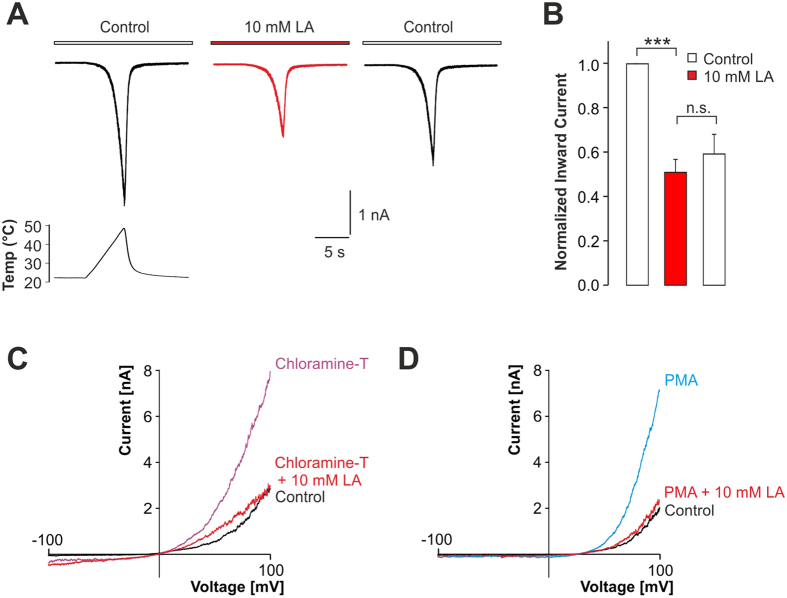
Inhibition of heat- and pro-algesic-evoked TRPV1 inward currents by LA. (**A**) Representative traces of inward currents evoked by heat ramps from ~23 °C to ~43 °C in control solution (left trace), in presence of 10 mM LA (middle trace) and after 1 min washout of LA (right trace). (**B**) Mean inward currents of heat-evoked TRPV1 currents normalized to control currents before LA application. Data are presented as mean ± S.E.M. Statistical differences are indicated by ***p < 0.001. (**C,D**) Membrane currents of TRPV1 monitored during 500-ms long voltage ramps from −100 mV to +100 mV in presence of control solution and after treatment with 500 μM chloramine-T (**C**) or 1 μM PMA (**D**) before and after application 10 mM LA.

We also examined the effects of LA on TRPV1 when activated by further endogenous agonists, including heat, oxidation and protein kinase C-mediated phosphorylation. 10 mM LA inhibited heat-evoked inward currents by 49 ± 6% (p < 0.001 paired t-test; n = 9) when hTRPV1 was activated at neutral pH 7.4 by heat ramps ranging from 23 °C to 48 °C (Fig. 3A,B). This LA-induced inhibition was partly irreversible. However, 10 mM LA did not seem to alter the temperature-threshold for activation of TRPV1 (data not shown). Oxidation of TRPV1 was achieved by application of the strong oxidant chloramine-T (500 μM for ~120 s), resulting in a strong potentiation of TRPV1-mediated outwardly rectifying membrane currents monitored during voltage ramps (Fig. 3C). When 10 mM LA was applied on cells which had been already oxidized, we observed a rapid and complete inhibition of the potentiated current (99% inhibition, n = 5, Fig. 3C). A similar approach was undertaken with the PKC-activator PMA (Phorbol 12 myristate 13 acetate, 1 µM), which also induced a prominent potentiation of TRPV1-mediated membrane currents monitored at positive potentials (Fig. 3D). When 10 mM LA was applied, this potentiation was reduced by 94% (Fig. 3D, n = 7).

### Lactate inhibits proton-evoked calcium-influx through recombinant TRPV1 channels as well as in cultured mouse DRG neurons

**Figure 4:**
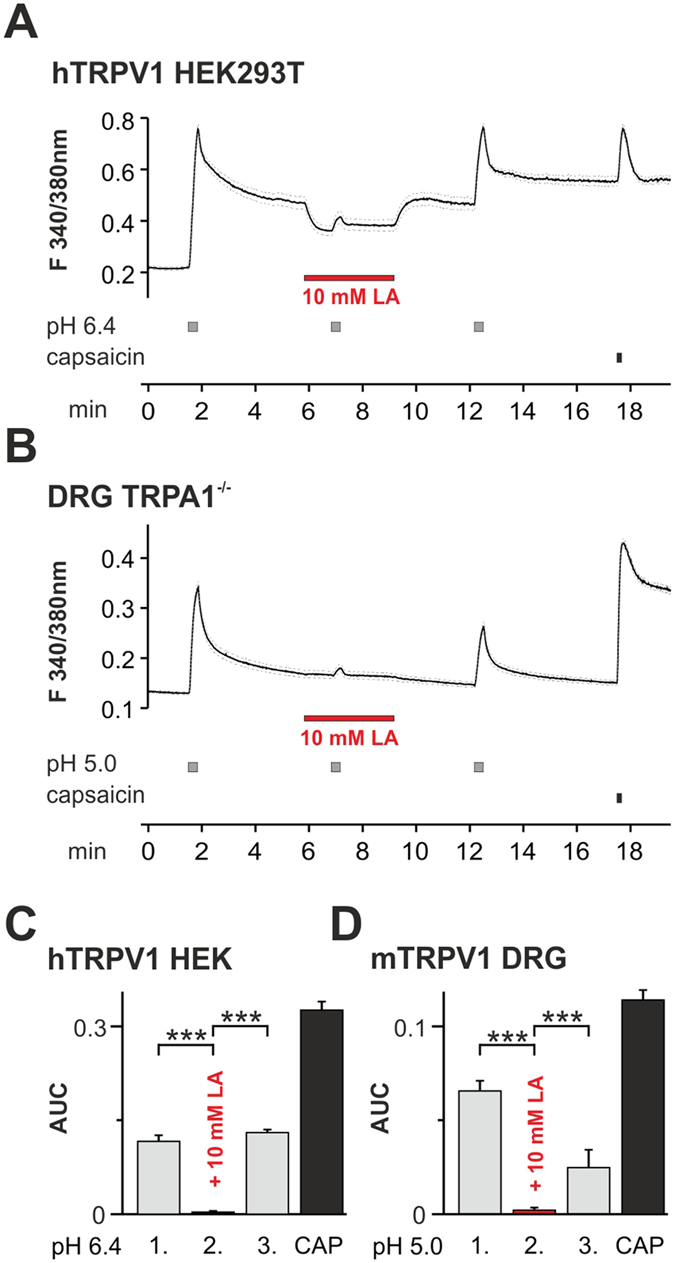
Inhibition of TRPV1-mediated proton-evoked Ca^2+^-influx by LA. (**A**) Average effects of 10 mM LA on Ca^2+^-influx induced by pH 6.4 in cells expressing hTRPV1 as determined by ratiometric imaging. Cells were challenged with 3 subsequent 20s long applications of pH 6.4 within intervals of 5 min. 10 mM LA was co-applied with pH 6.4 during the second application. Capsaicin (0.3 μM) was applied to confirm expression of TRPV1. Dashed lines represent standard error of the mean. (**B**) Average effects of 10 mM LA on Ca^2+^-influx induced by pH 5.0 in DRG neurons from TRPA1^−/−^ knockout mice; same protocol as in (**A**). (**C**,**D**) Average responses to the 3 applications of pH 6.4 on hTRPV1 (**C**) and pH 5.0 on DRG neurons (**D**) expressed as area under the curve for increase in intracellular calcium calculated for all capsaicin-responsive cells. Data are presented as mean ± S.E.M. Statistical differences are indicated by ***p < 0.001.

Since TRPV1 permeates calcium ions, we next employed ratiometric [Ca^2+^]_i_ measurements on hTRPV1 expressing HEK293T cells, to confirm the reduction in TRPV1 conductivity. Fura2-AM loaded cells were challenged by 3 consecutive 20 s lasting applications of an acidic solution (pH 6.4) at intervals of 5 minutes. As is demonstrated in Fig. 4A, application of 10 mM LA was started 1 minute before and until 2 minutes after the second pH 6.4 stimulation. It reversibly inhibited the increase in intracellular calcium concentration. We subsequently stimulated the same cell by 0.3 μM capsaicin to verify the expression of TRPV1.

As functional properties of overexpressed TRPV1 channels in HEK293T cells might not fully resemble those of TRPV1 channels in native neurons, we also examined the effect of 10 mM LA on proton-responses of TRPV1 in cultured mouse dorsal root ganglion (DRG) cells. As LA was previously demonstrated to activate TRPA1 in mouse DRG neurons^15^, we therefore used DRG neurons derived from TRPA1-deficient mice. In order to obtain robust responses in mouse DRG neurons expressing considerably lower levels of TRPV1 as compared to transfected HEK293T cells, proton-responses were elicited by pH 5.0. When performing the same protocol as described for hTRPV1 expressing HEK293T cells, we observed that 10 mM LA effectively inhibited proton-evoked calcium influx also in DRG neurons expressing TRPV1 (Fig. 4B). Proton activation was almost abolished by 10 mM LA both in HEK293T cells (ANOVA F(2.636) = 128.0; *p < 0.001 each; Fig. 4C) and in DRG neurons (ANOVA F(2.531) = 78.6; *p < 0.001 each; Fig. 4D).

### Lactate inhibits TRPV1-mediated proton-evoked release of CGRP from mouse nerves

**Figure 5:**
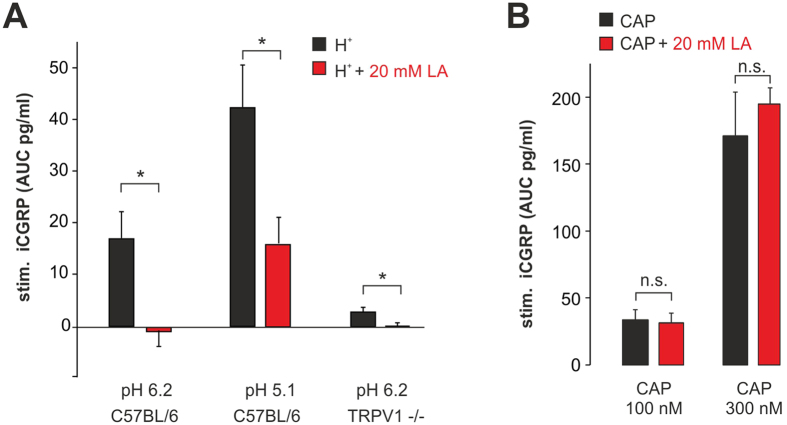
LA inhibits proton-evoked CGRP-release from sciatic nerves. (**A**) Mean CGRP release evoked by pH 6.2 and pH 5.1 without and with 20 mM LA in nerves from wild type (C57BL/6) and TRPV1-knockout mice (TRPV1^−/−^). (**B**) Mean CGRP-release evoked by 100 and 300 nM capsaicin (CAP) without and with 20 mM LA at pH 7.4. Average stimulated release of CGRP as measured by CGRP ELISA (stim. iCGRP) expressed as pg/ml. Data are presented as mean ± S.E.M. Statistical differences are indicated by *p < 0.05 and n.s. (not significant).

Activation of TRPV1 in sensory axons results in a prominent release of the neuropeptide CGRP, and proton-evoked release of CGRP from mouse sciatic nerves crucially depends on the expression of TRPV1^17^. Thus, we also examined the effect of LA on CGRP release induced by protons and capsaicin on isolated mouse nerves. As expected, the application of LA per se (20–60 mM; pH 7.4) did not induce any release of CGRP from isolated nerves (data not shown). However, 20 mM LA strongly inhibited CGRP release from nerves induced by both pH 6.2 and 5.1 (n = 5, p = 0.04, n = 6, p = 0.04 both Wilcoxon matched pairs test, respectively; Fig. 5A). The role of TRPV1 for pH 6.2-evoked CGRP release was additionally examined on nerves from TRPV1-knockout mice. In nerves lacking TRPV1, proton-evoked CGRP-release was significantly smaller as compared to the release in nerves from wild type C57Bl/6 mice (n = 5; p < 0.01 U-test). 20 mM LA still induced a significant TRPV1-independent inhibition of this pH 6.2-evoked CGRP release in TRPV1 knockout nerves (n = 8 each, p = 0.02 U-test; Fig. 5A), which indicates that other proton-sensitive transduction molecules are inhibited by LA as well. In contrast to the strong suppression of the pH induced CGRP release, LA did not affect CGRP release induced by 100 nM (n = 4 each group) or 300 nM (n = 8 each group) capsaicin in nerves from wild type mice (Fig. 5B; p > 0.05 Wilcoxon matched pairs test).

### Lactate modulates gating of TRPV1

**Figure 6:**
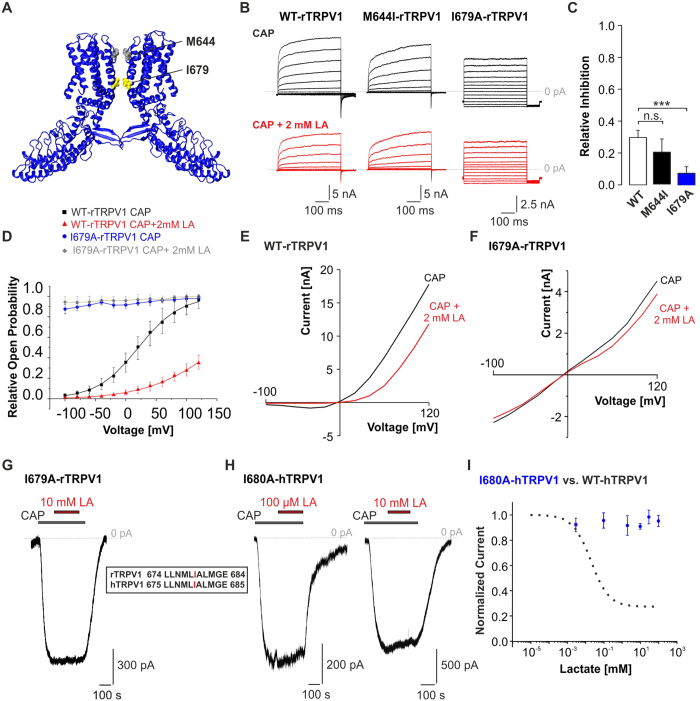
LA reduces the open probability of the TRPV1 lower gate. (**A**) Cryo-electron microscopy structure of the rTRPV1 channel (in blue chain A and D) derived and modificated from pubmed 3J5p.pdb and sequence gi71164787. M644 in the selectivity filter of rTRPV1 is shown in grey, and I679 located in the lower gate of the channel pore in yellow. (**B**) Representative recordings of WT, M644I- or I679A-rTRPV1 currents evoked by 500 ms long steps from −100 to +140 mV in presence of capsaicin or capsaicin +2 mM LA. Note that the current kinetic of mutation I679A-rTRPV1 is dramatically changed and shows no inhibition by 2 mM LA. The dashed line represents 0 pA current. (**C**) Mean relative inhibition of WT, M644I- and I679A-rTRPV1 by 2 mM LA at +120 mV. I679A-rTRPV1, but not M644I-rTRPV1 shows a significantly reduced inhibition as compared to the WT-rTRPV1. (**D**) Voltage dependence of relative open probabilities of WT-rTRPV1+CAP (black) and mutant I679A-rTRPV1+CAP (blue) in absence of LA and WT-rTRPV1+CAP+LA (red) and mutant I679A-rTRPV1+CAP+LA (grey) in presence of 2 mM LA. Activation curve of WT-rTRPV1 is declined and shifted towards positive potentials in presence of LA. Open probabilities of I679A-rTRPV1 are voltage insensitive, indicating a constitutively opened lower gate (**E**,**F**). Membrane currents of WT-rTRPV1 (**E**) and I679A-rTRPV1 (**F**) monitored during 500 ms long voltage steps from −100 mV to +120 mV in presence of 50 nM capsaicin or capsaicin +2 mM LA. (**G**,**H**) Representative current traces of I679A-rTRPV1 (**G**) and I680A-hTRPV1 (**H**) challenged by 50 nM capsaicin in combination with 100 μM or 10 mM LA. (**I**) Dose-response curves for LA–induced inhibition of capsaicin-evoked inward currents of hTRPV1-WT (black dashed line; compare Fig. 2B) and hTRPV1-I680A (blue). The solid lines represent fits with the Hill equation (mono-exponential Hill-4 parameter logistic). Data are presented as mean ± S.E.M. Statistical differences are indicated by ***p < 0.001.

To gain more insights into the mechanism of LA-induced inhibition of TRPV1, we investigated whether gating of TRPV1 is affected by LA. We focussed on two hydrophobic amino acids which have been postulated as being part of the upper and lower gate of TRPV1^21^,^22^ (Fig. 6A). M644 is located in the selectivity filter, which is accomplished in the open state of the upper gate^21^. The mutation M644I was reported to decrease the permeability of NMDG for rat (r) TRPV1^23^. I679 is located in the S6 segment of rTRPV1 protein and is suggested to be part of the lower gate^21^,^22^. We first compared the voltage-dependency of capsaicin-induced currents in presence and absence of 2 mM LA, which is sufficient to induce a prominent reduction of hTRPV1 currents (Fig. 2). The voltage protocol consisted of 500 ms long steps between −100 mV and +140 mV every 20 mV, followed by a 100 ms test pulse to −100 mV for comparison of tail current amplitudes. Wild type rTRPV1 and M644I-rTRPV1 showed a time-dependent increase of the current amplitude at positive potentials (Fig. 6B). In contrast, I679A-rTRPV1 did not display this time-dependent gating. Application of 2 mM LA reduced the current amplitude of WT-rTRPV1 (30 ± 5%, n = 6) and M644I-rTRPV1 (20 ± 8%, n = 6) with a similar potency (p > 0.05, n = 6), but left I679A-rTRPV1 largely unaffected (n = 4, Fig. 6C). We also determined the relative open probabilities of rTRPV1 at the end of the preceding voltage steps by exploring tail current amplitudes obtained at −100 mV (Fig. 6D). While WT-rTRPV1 showed a strong increase of the relative open probability between −100 and +120 mV in the absence of LA, 2 mM LA dramatically decreased the open probabilities and shifted the activation curve by about 75 mV towards positive potentials (control: V_1/2_ = +8 ± 1 mV, n = 5; 2 mM LA: V_1/2_ = +83 ± 8 mV, n = 5, p < 0.01). In contrast, I679A-rTRPV1 displayed no voltage-dependent change in open probability, indicating that capsaicin constitutively opens the lower gate of the mutant TRPV1 channel. In line with these results, the IV-curves of whole-cell currents of WT-rTRPV1 displayed a prominent inhibition of inward and outward currents by 2 mM LA (Fig. 6E). In contrast, I679A-rTRPV1 displayed a linear current to voltage relationship with a lack of inhibition by 2 mM LA (Fig. 6F). Likewise, 10 mM LA failed to inhibit capsaicin-evoked inward currents of I679A-rTRPV1 (Fig. 6G, n = 6). A sequence alignment between rTRPV1 and hTRPV1 revealed that I679 in rTRPV1 corresponds to I680 in hTRPV1 (Fig. 6G). In order to substantiate the role of this residue for LA inhibition, we finally explored the influence of LA on I680A-hTRPV1. As is demonstrated in Fig. 6H,I, both low and high concentrations of LA completely failed to inhibit capsaicin-induced inward currents (n = 4–7 for each concentration). Furthermore, 10 mM LA also failed to inhibit inward currents of I680A-hTRPV1 evoked by pH 5.4 (data not shown).

### Lactate affects TRPV1 from the extracellular side

**Figure 7:**
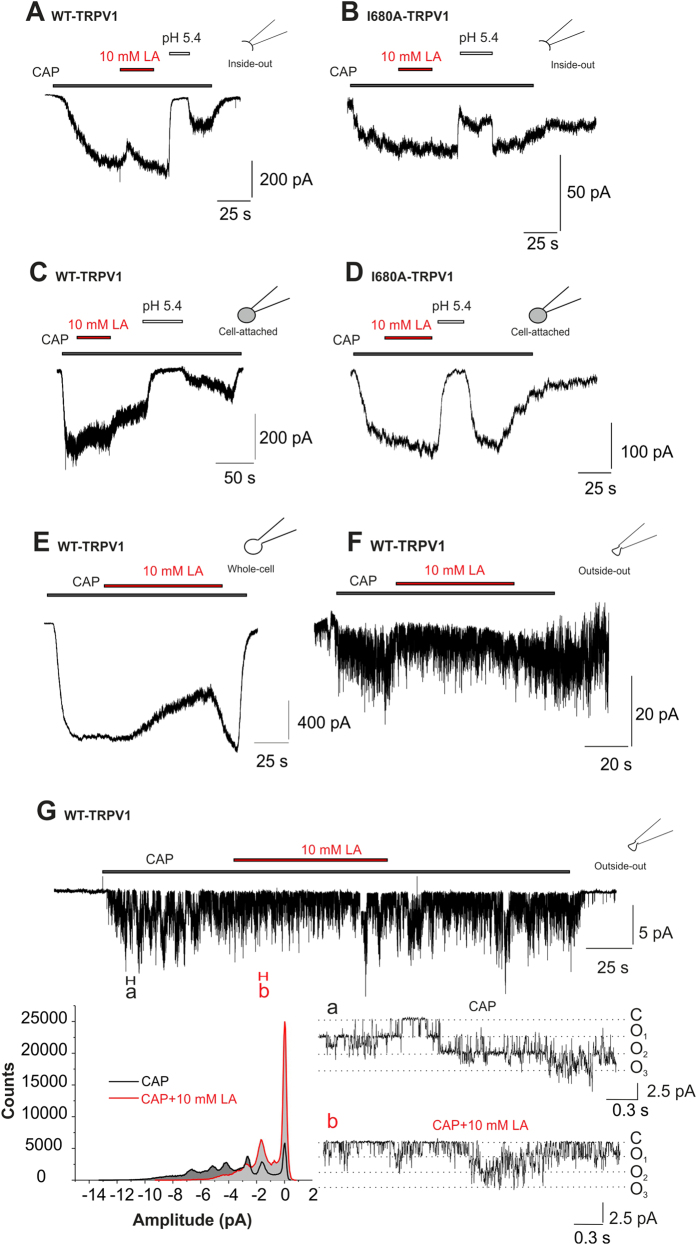
LA inhibits TRPV1 by an extracellular site of action. (**A**,**B**) Representative current traces from inside-out patches of WT-hTRPV1 (**A**) and I680A-hTRPV1 (**B**) challenged with capsaicin (500 nM) alone or in combination with 10 mM LA or pH 5.4. While 10 mM LA fails to inhibit both WT- and I680A-hTRPV1 in this mode, pH 5.4 induces as rapid and reversible inhibition. (**C**,**D**) Representative current traces from cell-attached recordings of WT-hTRPV1 (**C**) and I680A-hTRPV1 (**D**) challenged with capsaicin (500 nM) alone or in combination with 10 mM LA or pH 5.4. While 10 mM LA fails to inhibit both WT- and I680A-hTRPV1 in this mode, pH 5.4 induces as rapid and reversible inhibition due to intracellular acidosis. (**E**,**F**) Representative current traces from whole cell (**E**) and outside-out (**F**) WT-TRPV1 currents of the same cell, challenged with 50 nM capsaicin ±10 mM lactate at −60 mV. In both cases, we can see a clear inhibition of capsaicin-induced currents by lactate. (**G**) Single channel recordings in outside-out configuration at −30 mV (upper trace) reveal an increased open probability of TRPV1 channels upon capsaicin application (**a**) which is shifted back to the closed state by lactate (**b**). Sections a,b represent 300 ms of the upper trace with an expanded time scale (a, black, 200 nM CAP; b, red, 200 nM CAP+10 mM LA). The amplitude histogram (left bottom) displays a lactate inhibition of capsaicin-induced events leading to an increase in the closed state of TRPV1 channels.

In order to elucidate whether LA inhibits TRPV1 from the extracellular or intracellular side, we finally compared the effects of 10 mM LA on capsaicin-induced currents under cell-attached and cell-free inside-out patch clamp conditions. In contrast to the prominent inhibition by 10 mM LA in the whole cell configuration, the application of 10 mM LA on capsaicin-evoked currents in cell-free inside-out configuration did not show any inhibition for WT-TRPV1 (Fig. 7A, n = 5). We sometimes observed a transient perfusion artefact at the beginning of the LA-application that disappeared within 5–10 s.

Intracellular acidification has been demonstrated to inhibit TRPV1^18^ (Fig. 7A). Therefore the missing LA inhibition might be interpreted as secondary effect on TRPV1 channels through an intracellular acidification that is obviously absent in inside-out patches. To clarify this hypothesis we referred to mutation I680A of human TRPV1 which showed no LA-inhibition in whole cell-experiments. If an intracellular acidification is responsible for TRPV1 inhibition, mutation I680A should be insensitive to decreasing internal pH. However, we found a sustained reduction of I680A-hTRPV1 currents in inside-out patches when pH 5.4 was applied to the intracellular side, while LA did not affect the channel (Fig. 7B, n = 3). This finding implicates that block of TRPV1 by LA employs a mechanism distinct from inhibition by intracellular acidosis.

Furthermore, we performed experiments in the cell-attached configuration where the access of externally applied LA to the recorded channels is omitted by the tight seal of the pipette tip. In cell-attached recordings on WT-TRPV1 (Fig. 7C, n = 6) and on mutant I680A-TRPV1 (Fig. 7D, n = 8), we observed a potent reduction of the capsaicin-induced currents by pH 5.4, but a lack of inhibition for both constructs by LA. These data confirm that TRPV1 is inhibited by protons from the intracellular side, and by LA from the extracellular side.

Given that most experiments of the manuscript were carried out in whole cell configuration, where agents of the bath solution have direct access to the extracellular surface of the membrane, we consulted whole cell measurements (Fig. 7E) which were subsequently followed by recordings from the excised outside-out patches (Fig. 7F) of the same cell. In both cases we saw an inhibition by 10 mM lactate. Additionally, we aimed to reduce the patch size to obtain single channel events in the outside-out configuration. These recordings (Fig. 7G, n = 6) revealed a high probability for TRPV1 channels to reside in the open state after application of capsaicin (Fig. 7G, section “a”). 10 mM LA inhibited the capsaicin-induced openings and led to an increased closed state probability (Fig. 7G, section “b” and histogram), which perfectly fits to the shift of the activation curve of TRPV1 channels as shown in Fig. 6D. In summary, these data prompt to an extracellular site of lactate interaction to induce the inhibition of TRPV1 currents.

## Discussion

In this study we demonstrate a dose-dependent inhibition of the nociceptive transduction molecule TRPV1 by extracellular lactate. Systemic concentrations of LA exceeding 10 mM are commonly observed in patients suffering from major ischemic events like myocardial infarction or mesenterial ischemia. The high concentrations of LA found to be effective in this study have been shown to accumulate in tissue during ischemia in particular in working muscle. Bangsbo and colleagues reported that up to 50 mM LA can be measured in muscle tissue during excessive exercise^10^,^11^,^12^, and up to 90 mM LA was described to accumulate in tissues following cerebral ischemia^24^. We found that LA at concentrations considerably lower than the physiological normal plasma level of 2 mM inhibits TRPV1 as well. This finding suggests that LA might be an important endogenous regulator of TRPV1 both under physiological as well as pathophysiological conditions. Bearing in mind that our data were obtained by *in vitro* experiments, we can only speculate about the role of lactate as endogenous inhibitor of TRPV1. At this stage however, we are not aware of any mouse model suitable to corroborate our findings *in vivo*. Saying this, it is worthy to note that the role of TRPV1 as the principle proton-sensor in sensory neurons lack evidence from *in vivo* studies as well^2^.

We demonstrate inhibitory properties of LA on both heterologously expressed TRPV1 channels as well as TRPV1 endogenously expressed in mouse DRG neurons. In a more intact preparation of isolated peripheral nerves, LA also effectively inhibited TRPV1-mediated neuropeptide release when challenged by acidosis. Thus, LA-induced inhibition of TRPV1 was effective throughout all assays performed in this study. Moreover, we sought to identify the mechanisms by which LA affects TRPV1. As protons activate TRPV1 mainly by interaction with two extracellular glutamate residues^25^ and capsaicin via the intracellular located vanilloid-binding domain^26^, it seemed unlikely that LA inhibits TRPV1 in a competitive manner by interacting with both agonist binding sites. Notably, activation of the related irritant channel TRPA1 by weak acids (including LA) was suggested to be due to intracellular acidosis^15^. As a weak acid, mainly the protonated form of LA can permeate the membrane to induce intracellular acidosis. In neurons, this process seems to additionally involve the neuronal monocarboxlyate transporter 2 where the anionic form of lactate and a proton are transported by a symport mechanism through the membrane^27^. Indeed, TRPV1 has been demonstrated to be inhibited by intracellular acidosis^18^. Additionally, more recent studies have shown that protons inhibit permeation of cations through TRPV1 in a voltage-dependent and concentration-dependent manner by binding acidic residues inside or near the conducting pore^19^,^20^. Therefore, intracellular acidosis seemed like a plausible mechanism for LA-induced inhibition of TRPV1. However, while our findings confirm that TRPV1 is inhibited by intracellular protons, LA-induced inhibition seems to be accomplished through an independent extracellular mechanism.

We show that LA inhibits TRPV1-mediated membrane currents not only during acidosis, but also at neutral pH-values, e.g. activation by capsaicin or heat at pH 7.4. With a pKa of 3.85 the membrane permeable fraction of LA is almost negligible at pH 7.4. Indeed, a study on isolated rat ventricular cardiac myocytes revealed that the high concentration of 2–10 mM LA only induced a minute shift (<0.3 units) of the intracellular pH-value^28^,^29^. However, inhibition of TRPV1 by intracellular acidosis requires considerably lower pH values (<pH 6)^18^. Furthermore, we observed strong inhibition of TRPV1 by LA despite buffering the intracellular solution with 10 mM HEPES. Another finding, which argues against intracellular acidosis as main mechanism for LA inhibition, is the selective inhibition of mutant I680A-hTRPV1 by protons and not by LA in the inside-out configuration of patch clamp recordings. As LA inhibits only WT but not the mutant I680A-TRPV1 in whole cell recordings, we conclude that intracellular acidosis and LA inhibit TRPV1 via distinct mechanisms. Our findings on cell-attached experiments confirm the results from inside-out recordings. By conducting outside-out patches after performing lactate inhibition in whole cell configuration directly at the same cell, we could furthermore support the notion that LA modulates TRPV1 from the extracellular side. Moreover, lactate-inhibition of capsaicin-induced events leads to an increase in the closed state of TRPV1 channels by lactate.

The limited membrane permeability of LA at neutral pH might also explain the discrepancy between LA inhibition of CGRP-release induced by acidosis (Fig. 5A; pH 5.1 or 6.2) and the missing LA effect on capsaicin-evoked CGRP-release at pH 7.4 (Fig. 5B). For LA to reach the axonal membrane and thus TRPV1 channels, it would have to permeate through the perineurial barrier. Acidosis renders LA membrane permeable, because diffusion of the protonated and uncharged lactic acid is increased by about 100 fold (following Henderson-Hasselbalch equation lactic acid concentration is 0.03% at neutral pH and 3% at pH 5.4) which should significantly increase passive diffusion. Furthermore an increased lactate uptake is favoured by acidification through pH dependent monocarboxylate transporters 2^30^,^31^. Accordingly, this effect might explain the inhibition of proton-evoked CGRP-release. However only a minimal fraction of LA might rapidly permeate this barrier at pH 7.4, and therefore LA failed to inhibit CGRP release at neutral pH.

When approaching the mechanism of LA-inhibition, the first finding pointing to the channel gating as a possible interaction site of LA came from the experiments demonstrating that LA induced a stronger inhibition of inward currents at −100 mV as compared to outward currents at +100 mV (Figs 1D and 2D). The same pattern was reported for TRPV1 inhibitors like QX-314 and tetraethylammonium (TEA)^32^,^33^. Moreover, a shift of the activation curve confirms that current reduction in presence of LA is caused by a diminished TRPV1 open probability (Figs 6D and 7G). Accordingly, the shift of the activation curve fully explains the asymmetric effect on negative vs. positive potentials. Finally, we found that a replacement of the lower gate amino acid I680 in hTRPV1 (I679 in rTRPV1) abolished LA-induced inhibition. In contrast, we could not determine any effect on LA-induced inhibition by the replacement of M644 in the upper gate of TRPV1. In line with these results, the mutant construct TRPV1-I679A still requires capsaicin to be opened due to an intact upper gate. Although these data do not reveal exactly how LA inhibits TRPV1, it is likely that LA induces conformational rearrangements in the TRPV1 protein that reduce the lower gate open probability.

Coming to the physiological relevance of LA-inhibition of TRPV1, a recently established rat model of thrombus-induced ischemic pain suggests that mechanical hyperalgesia is mainly driven by ASICs and P2X receptors, but not by TRPV1^34^. This observation correlates well with our observations and the fact that the acid-sensitivity of ASICs is potentiated by LA and indirectly by ATP via activation of the purinergic P2X5 receptor^35^. Furthermore, pain induced by ischemia also seems to depend on TRPA1^36^. This notion correlates well with the role of TRPA1 as the predominant receptor for weak acids and for hypoxia in rodent sensory neurons^15^,^37^. Our finding that TRPV1 is inhibited by physiological as well as pathophysiological concentrations of LA fully corroborates these reports. Accordingly, the expression of ASICs, TRPA1 and TRPV1 in a multimodal nerve terminal could help to respond to the diversity of nociceptive stimuli by selecting the adequate transduction molecules. Consequently, our study unravels that TRPV1 inhibition by LA during ischemia prevents the multimodal nerve terminal from further depolarization.

Taken together, our data demonstrate for the first time that TRPV1 distinguishes between acidosis as opposed to lactic acidosis, and postulates a yet unknown role for LA as an endogenous regulator of TRPV1.

## Materials and Methods

### Cell culture and transfection

HEK293T cells were transiently co-transfected with 4–5 μg cDNA of rat TRPV1 (rTRPV1) identical to NM_031982.1 in pcDNA3 (a kind gift from Dr. David Julius, San Francisco, CA, USA) or human TRPV1 (hTRPV1) identical to NM_080706.3 and subcloned in pcDNA3.1(+), and 2 μg pEGFP (Clonetech, Palo Alto, USA) by using calcium phosphate precipitation. Cells were cultured in standard DMEM (D-MEM, Gibco, BRL Life Technologies, Karlsruhe Germany) with 10% FBS (Biochrom, Berlin Germany), 100 U/ml penicillin and 100 μg/ml streptomycin (Gibco, Karlsruhe, Germany) and 2 mM Glutamax (Gibco, Karlsruhe Germany) for optimal growth conditions. For stably transfected hTRPV1-HEK293T cells, (cDNA in pJTI ^TM^R4 Int, a kind gift from Dr. Peter Zygmunt, Lund, Sweden) medium was supplemented with 5 μg/ml Blasticidin (PAA, Pasching, Austria) and 0.35% Zeocin (Invitrogen, Toulouse, France). The stable TRPV1-cell line was inducible by addition of 0.1 μg/ml tetracycline (Sigma-Aldrich, Germany) 16 to 24 hours prior to measurement. Cells were cultivated at 37 °C and 5% CO_2_ and dissociated in petri dishes 12–24 h before starting the experiments.

### Mutagenesis

Using site-directed mutagenesis kit (QuikChange Site-directed-Mutagenesis Kit, Agilent Technologies, CA, USA) and PfuTurbo DNA-Polymerase (Agilent Technologies, CA, USA) we introduced lower gate mutation I680A into human TRPV1-pcDNA3.1 (+). The resultant construct I680A-hTRPV1 is homolog to I679A-rTRPV1 (a kind gift from Dr. Viktorie Vlachová, Prague, Czech Republic). Mutagenesis was designed by use of Vector NTI^®^Software (Thermo Fisher Scientific, MA, U.S.A), vector cDNA was linearized with BstEII and EcoRI and single stranded template cDNA was synthesized from T7 priming site. Mutant constructs were verified by standard didesoxynucleotide sequencing (GATC Sequencing, GATC Biotech AG, Cologne, Germany). Selectivity filter mutant construct M644I-rTRPV1 was kindly provided by Dr. Michael J. Caterina (Baltimore, Maryland, USA).

### Electrophysiology

Standard patch clamp recordings were performed in whole cell, cell attached and inside-out configuration using an HEKA Electronics USB 10 amplifier and the Patchmaster Software (HEKA Electronics, Lambrecht, Germany) or an Axopatch 200B amplifier and pClamp10.5 Software (Molecular Device, Sunnyvale CA, USA). For whole cell recordings and macropatches borosilicate pipettes with a resistance of 3–5 MΩ were pulled with a laser puller (Sutter Instrument, Model P-2000, Novato, CA, USA). The standard intracellular solution contained (in mM): KCl (140), MgCl_2_ (2), EGTA (5), HEPES (10) with pH adjusted to 7.4 by KOH. Standard calcium free extracellular solution contained (in mM): NaCl (140), KCl (5), MgCl_2_ (2), EGTA (5), HEPES (10) and glucose (10); pH 7.4 adjusted by sodium hydroxide (NaOH). The osmolarity of solutions was set to 277 mOsm intracellular and 304 mOsm extracellular. For proton-evoked currents (acidic pH solutions) we used 10 mM 2-(N-morpholino)ethanesulfonic acid (=MES) as buffering solution instead of HEPES (10 mM) in the bath and pipette solution. Excised patch recordings were performed using the inside-out patch clamp configuration with internal solution in the bath and external solution in the pipette.

Transfected cells were visualized by EGFP-fluorescence prior to the recording. Only cells yielding an initial GΩ-seal and less than 50 pA leak current at −60 mV holding potential were included for further analysis. Membrane currents were low pass filtered at 2 kHz and digitized with a sampling rate of 5–10 kHz (Digidata 1550, Molecular Devices, Sunnyvale CA, USA). Drugs were applied by a gravity driven perfusion system allowing a focal application of the test solution within a distance of <100 μm from the cell. To accomplish comparable conditions for each experiment after drug application, only one cell was recorded per dish.

Temperature of the applied test solutions was controlled with a perfusion system incorporating a rapid-feedback temperature control allowing rapid heating or cooling^38^. The patchmaster/fitmaster software (HEKA Electronic) was used for acquisition of temperature and current data and for off-line analysis.

Data analysis was performed by a combination of Fitmaster (HEKA Electronics, Lambrecht, Germany) or Clampfit 10.5 software (Axon, Molecular Devices, Sunnyvale CA, USA) and Origin 8.5.1 (Origin Lab, Northampton, MA, USA). For statistical analysis of multiple samples ANOVA following LSD post hoc test was utilized. The relative inhibition of proton or capsaicin induced currents was calculated by relative inhibition = 1 − I/I_0_; where I = current in presence of LA, I_0_ = current in absence of LA. The dose-response curve was fitted with a mono-exponential fit (Hill-4 parameter logistic) and normalized currents were assessed by I/I_0_. For the calculation of the relative open probabilities we measured the instantaneous tail current amplitudes obtained at the test potential of −100 mV at the end of the preceding voltage steps and normalized these currents to Imax (the maximal negative current amplitude of the appropriate Boltzmann fit).

### Preparation of DRG of TRPA1/TRPV1 knockout mice

Animal care and treatment were conducted according to IASP-guidelines and all procedures of this study were approved by the animal protection authorities (District Government Mittelfranken, Ansbach, Germany). Adult (8–12 weeks) TRPA1-knockout mice (TRPA1^−/−^) and TRPV1-knockout mice (TRPV1^−/−^) with C57BL/6 background were used. Original breeding pairs of TRPA1^−/−^ and TRPV1^−/−^ were gifts from Dr. John B. Davis (Department of Neuroscience Research, SmithKline Beecham Pharmaceuticals, Harlow, UK) and Dr. David Corey (Harvard University, Boston, USA) and continuously backcrossed to C57BL/6. All animals were genotyped prior to experiments. Animals were sacrificed in pure CO_2_ atmosphere. Dorsal root ganglion (DRG) cells from all spinal levels were excised, transferred into Dulbecco’s modified Eagle’s medium solution (DMEM, GIBCO-Invitrogen, Germany) containing 50 μg/ml gentamicin (Sigma-Aldrich, Germany) and incubated in 1 mg/ml collagenase (Sigma type XI) and 0.1 mg/ml protease (Sigma) for 40 min at 37 °C. The ganglia were then gently dissociated using a fire-polished silicone-coated Pasteur pipette and neurons were plated onto borosilicate glass coverslips which had been coated with poly-L-lysine (0.2 mg/ml for 30 min, Sigma-Aldrich, Germany). Cells were cultured in serum-free TNB-100 basal medium supplemented with TNB 100 lipid-protein complex, 100 U/ml streptomycin and 100 μg/ml penicillin (all Biochrom, Germany) at 37 °C and 5% CO_2_. Calcium imaging experiments were performed within 20–30 h after dissociation.

### Ratiometric [Ca^2+^]_i_ measurements

Either hTRPV1-expressing HEK cells or DRG neurons from TRPA1 knockout mice were stained by 3 μM fura-2-AM and 0.01% pluronic for about 45 min. Following wash out to allow fura-2-AM de-esterification, coverslips were mounted on an inverse microscope with a 20x objective (Axio observer D1, Zeiss). Using a software controlled 7-channel gravity-driven common-outlet superfusion system, coverslips were constantly superfused with a Ca^2+^-containing extracellular solution (in mM): NaCl (145), KCl (5), CaCl_2_ (1.25), MgCl_2_ (1), Glucose (10), Hepes (10). Fura-2 was excited using a microscope light source and an LEP filter wheel (Ludl electronic producs Ltd.) to switch between 340 and 380 nm excitation wavelengths. Time dependent changes in Fura-2 fluorescence were acquired at 510 nm from a series of images. Images were exposed for 20 and 40 ms (HEK cells) or 20 ms and 10 ms (DRGs) and acquired at a rate of 1 Hz with a CCD camera (Cool SNAP EZ, Photometrics). Data were recorded using VisiView 2.1.1 software (all from Visitron Systems GmbH, Puchheim, Germany). Background fluorescence was subtracted before calculation of ratios fluorescence ratios (F340/380 nm) for region of interest. Changes of the area under curve (AUC, delta ratio F340/380 nm vs. time) represent relative changes in calcium concentrations. A capsaicin (0.3 μM) stimulus was used to identify TRPV1 expressing cells on a functional level and ionomycin (5 μM) or potassium chloride (60 mM) were applied to induce maximum calcium influx as a control at the end of each experiment in HEK cells or DRG neurons. Statistical analysis of multiple groups ANOVA following HSD post hoc test was calculated.

### Neuropeptide release

The sciatic nerves were harvested from adult wild type C57Bl/6 or TRPV1 knock-out mice after sacrificing mice in a pure CO_2_ atmosphere (approved by the Animal Protection Authority, District Government Mittelfranken, Ansbach, Germany). Nerves were excised from their origin at the lumbar plexus to their branching into tibial, sural, and peroneal nerves and then washed for 30 min in carbogen-gassed (95% O_2_ and 5% CO_2_) synthetic interstitial fluid (SIF) containing (in mM): NaCl (108), KCl (3.48), MgSO_4_ (3.5), NaHCO_3_ (26), NaH_2_PO_4_ (1.7), CaCl_2_ (1.5) sodium gluconate (9.6), glucose (5.5), and sucrose (7.6)^39^,^40^. Experiments were performed at 37 °C. After washout, the preparations were first incubated for two subsequent periods of 5 min in glass tubes containing SIF to determine basal CGRP release (Samples 1 and 2, S1 and S2). This was followed by 5-min incubation in tubes containing different test solutions (Sample 3, S3) to stimulate or inhibit CGRP-release and a final 5-min incubation period in SIF to assess reversibility of CGRP release (Sample 4, S4). The supernatant incubation fluid was recovered and stored on ice, and the CGRP content of the incubation fluid was measured using a commercial enzyme immunoassay kit (Bertin Pharma, Montingy le Bretonneux, France) with a detection limit of 2 pg/ml. The antibodies used are directed against human α/β-CGRP but are 100% cross-reactive against mouse CGRP. The enzyme immunoassay plates were analyzed photometrically using a microplate reader (Dynatech, Channel Islands, UK). Column diagrams reflect overall stimulated CGRP release (AUC, (S3 + S4)-(S1 + S2)). Within one experimental group, data were compared by the Wilcoxon matched pair test or between groups using the Mann-Whitney U test.

### Statistics

Data were depicted as mean values ± S.E.M and statistical significance was tested by the Student’s t-test or by ANOVA. Significance levels are indicated as n.s. (not significant), *p < 0.05, **p < 0.01 and ***p < 0.001.

### Chemicals

1 mM capsaicin (CAP, Biotrend, Cologne, Germany) was dissolved in ethanol and 1 M L-(+)-Lactic acid (LA, Sigma Aldrich, Munich, Germany) was dissolved in water. All stock solutions were stored at −20 °C and diluted into appropriate test concentrations in standard internal (inside-out patches) or external solution (whole cell, cell-attached and outside-out recordings) prior to the experiments. Final concentrations of LA (100–0.3 mM) were adjusted with NaOH to the appropriate pH. Solutions with lower LA concentrations (< 30 μM) did not change the pH of the standard solution.

## Additional Information

**How to cite this article**: de la Roche, J. *et al.* Lactate is a potent inhibitor of the capsaicin receptor TRPV1. *Sci. Rep.* **6**, 36740; doi: 10.1038/srep36740 (2016).

**Publisher’s note:** Springer Nature remains neutral with regard to jurisdictional claims in published maps and institutional affiliations.
